# Highly Diverse Shrub Willows (*Salix* L.) Share Highly Similar Plastomes

**DOI:** 10.3389/fpls.2021.662715

**Published:** 2021-09-03

**Authors:** Natascha D. Wagner, Martin Volf, Elvira Hörandl

**Affiliations:** ^1^Department of Systematics, Biodiversity and Evolution of Plants (With Herbarium), University of Goettingen, Göttingen, Germany; ^2^Biology Centre of the Czech Academy of Sciences, Institute of Entomology, Ceske Budejovice, Czechia

**Keywords:** *Chamaetia/Vetrix* clade, Eurasia, genome skimming, North America, phylogenomics, plastome evolution

## Abstract

Plastome phylogenomics is used in a broad range of studies where single markers do not bear enough information. Phylogenetic reconstruction in the genus *Salix* is difficult due to the lack of informative characters and reticulate evolution. Here, we use a genome skimming approach to reconstruct 41 complete plastomes of 32 Eurasian and North American *Salix* species representing different lineages, different ploidy levels, and separate geographic regions. We combined our plastomes with published data from Genbank to build a comprehensive phylogeny of 61 samples (50 species) using RAxML (Randomized Axelerated Maximum Likelihood). Additionally, haplotype networks for two observed subclades were calculated, and 72 genes were tested to be under selection. The results revealed a highly conserved structure of the observed plastomes. Within the genus, we observed a variation of 1.68%, most of which separated subg. *Salix* from the subgeneric *Chamaetia/Vetrix* clade. Our data generally confirm previous plastid phylogenies, however, within *Chamaetia/Vetrix* phylogenetic results represented neither taxonomical classifications nor geographical regions. Non-coding DNA regions were responsible for most of the observed variation within subclades and 5.6% of the analyzed genes showed signals of diversifying selection. A comparison of nuclear restriction site associated DNA (RAD) sequencing and plastome data on a subset of 10 species showed discrepancies in topology and resolution. We assume that a combination of (i) a very low mutation rate due to efficient mechanisms preventing mutagenesis, (ii) reticulate evolution, including ancient and ongoing hybridization, and (iii) homoplasy has shaped plastome evolution in willows.

## Introduction

Plastid markers are frequently used in plant phylogenetics because they possess several advantages over nuclear markers (Taberlet et al., 1991; Gitzendanner et al., 2018). They are haploid but occur in high copy number, which simplifies the sequencing process. Additionally, the availability of various plastid markers with different levels of molecular evolution combined with the conserved structure of the plastome makes them a popular choice for molecular systematic studies on different levels of divergence (Shaw et al., 2005, 2007; Wicke and Schneeweiss, 2015). Plastomes are usually inherited uniparentally and only rarely show recombination between differentiated plastid genomes (Wolfe and Randle, 2004; Bock et al., 2014). In combination with nuclear markers, this makes plastomes useful for the analysis of introgression, hybridization, and polyploidy. In addition, the dispersion of maternally inherited genomes occurs at shorter geographic distances than for nuclear genomes. The consequence of a reduced gene dispersal and high genetic drift in organelle genomes is a generally pronounced geographic structure (Besnard et al., 2011). However, despite all these advantages, single plastid markers have not been able to resolve phylogenetic relationships in some lineages due to a lack of informative sites (e.g., Percy et al., 2014). The advent of next generation sequencing techniques has enabled researchers to overcome this lack of information by analyzing complete plastomes at moderate costs, e.g., via genome skimming (Straub et al., 2012; Wicke and Schneeweiss, 2015). The number of available plastomes in databases that potentially might serve as reference for read mapping has drastically increased over the last years. Thus, plastome phylogenomics has been used in a broad range of studies, e.g., in rapidly radiating groups (Barrett et al., 2014; Straub et al., 2014) and lineages with high species diversity (Huang et al., 2014; Givnish et al., 2015; Nargar et al., 2018).

The genus *Salix* L. (Salicaceae) comprises about 400–450 species of trees and shrubs mainly occurring in the Northern Hemisphere (Fang et al., 1999; Skvortsov, 1999; Argus, 2010). Willows are ecologically and economically important, e.g., for biomass production (Smart et al., 2005; Karp et al., 2011), and they are considered as keystone plants for insect diversity (Narango et al., 2020). The reconstruction of the willow phylogeny has proven to be difficult based on traditional Sanger sequencing markers, which have failed to resolve interspecific relationships (Leskinen and Alström-Rapaport, 1999; Azuma et al., 2000; Chen et al., 2010; Savage and Cavender-Bares, 2012; Barcaccia et al., 2014; Percy et al., 2014; Lauron-Moreau et al., 2015; Wu et al., 2015). Based on morphological characters, the genus is divided into three (or five) subgenera: subgenus *Salix* s.l. (including subgenera *Salix* L., *Longifoliae* (Andersson) Argus*, Protitae* Kimura, or excluding the latter two), subgenus *Chamaetia* (Dumort) Nasarov in Kom., and subgenus *Vetrix* Dumort (Skvortsov, 1999; Argus, 2010; Lauron-Moreau et al., 2015; Wu et al., 2015). Recent studies have recommended that the latter two subgenera be merged into the *Chamaetia/Vetrix* clade (Wu et al., 2015; Wagner et al., 2018, 2020, 2021). This clade comprises about three quarters of the described species diversity in *Salix* containing more than 300 species classified in about 40 sections. Previous molecular studies based on traditional markers were able to confirm the monophyly of the genus and to separate a small, basal clade of subtropical to temperate trees (subg. *Salix* s.l.) (Leskinen and Alström-Rapaport, 1999; Azuma et al., 2000; Chen et al., 2010; Savage and Cavender-Bares, 2012; Barcaccia et al., 2014; Percy et al., 2014; Lauron-Moreau et al., 2015; Wu et al., 2015). Nevertheless, they failed to resolve the relationships among species of the diverse *Chamaetia/Vetrix* clade of shrub willows due to a lack of informative sites. Percy et al. (2014) tried to interpret the lack of variation in plastid barcoding markers with either coalescence failure and incomplete lineage sorting, or a selective, trans-specific sweep for a certain haplotype. The latter idea was supported by the observation of a non-random distribution of haplotypes and of polymorphisms within genes (Percy et al., 2014). However, the authors included only four plastid loci and focused mainly North American species. Selective sweeps were also hypothesized by Huang et al. (2014) to occur in plastomes of a few tested willow species. We aim to test if this pattern can be confirmed for complete plastome data in a more comprehensive sampling of species.

While single plastid or nuclear markers have failed to resolve relationships, recently, restriction site associated DNA (RAD) sequencing has been used to resolve relationships within the *Chamaetia/Vetrix* clade, rendering all taxonomic species as distinct monophyletic lineages (Wagner et al., 2018, 2020; He et al., 2021b). However, the data contained exclusively nuclear information. The availability of additional whole plastome data would increase our understanding of reticulate evolution within the genus. Reticulate evolution could involve several processes: ancient incomplete lineage sorting, horizontal gene transfer, and/or interspecific hybridization. In case of hybridization, including the hybrid origin of allopolyploids, the position of a species will differ between phylogenies that are based on plastid data representing the maternal lineage and nuclear data reflecting biparental inheritance. By analyzing plastomes in combination with nuclear data, it is thus possible to test hypotheses on reticulate evolution (Wicke and Schneeweiss, 2015) and to gain insight into the mode of origin for polyploids. Extant hybridization and introgression is an extensively reported and studied phenomenon in *Salix* and occurs even between distantly related species (Skvortsov, 1999; Argus, 2010; Hörandl et al., 2012; Gramlich et al., 2018). Additionally, ancient hybrid origin via allopolyploidy has been demonstrated for several European species (Wagner et al., 2020). Therefore, with the incorporation of plastid data, we may gain insights into whether frequent hybridization and chloroplast capture are leading to a spread of a few dominant plastid haplotypes as assumed among subg. *Chamaetia/Vetrix* (Percy et al., 2014; Lauron-Moreau et al., 2015). Chloroplast capture (Rieseberg and Soltis, 1991) often leads to a geographic clustering of haplotypes rather than a species-specific clustering. This is especially frequent in taxa with known hybridization and introgression (e.g., *Quercus*, Pham et al., 2017).

Recently, several single *Salix* plastomes were published (e.g., Lu et al., 2019; Wu et al., 2019; Chen, 2020) and the method of complete plastome sequencing was applied to the family Salicaceae s.l. to study phylogenetic relationships and diversification of five genera with a special focus on *Salix* and *Populus* (Huang et al., 2017; Zhang et al., 2018). This set was expanded by Li et al. (2019) to 24 species representing 18 genera. However, the authors focused on higher taxonomic levels and not on subgeneric relationships. Furthermore, few of the previous accessions covered the *Chamaetia/Vetrix* clade that contains most of the willow species. In this study, we present 41 complete plastomes of 32 *Salix* species, representing 19 out of circa 40 sections, with a specific focus on Eurasian species of the diverse *Chamaetia/Vetrix* clade to analyze plastome structure and variability. We combine the data with available *Salix* plastomes from Genbank to determine the utility of complete plastomes for phylogenetic analyses. The reconstructed relationships of the genus are used to examine if the taxonomical classification and/or biogeographical distribution are reflected by plastome data. We test whether selective sweeps could have shaped the plastome diversity of willows. Furthermore, we compare the plastome to nuclear RAD sequencing data in order to discuss ancient and recent hybridization and introgression in our target group. Finally, we discuss possible reasons that can explain the observed level of plastome variability within the genus *Salix*.

## Materials and Methods

### Plant Material

For this study, we sampled 32 species (41 accessions) representing 19 sections sensu Skvortsov (1999) (Table 1, Supplementary Table 1). Four species belonged to *Salix* subg. *Salix* s.l., (s. Skvortsov) and 28 species belonged to the shrub willow clade *Chamaetia/Vetrix*. Next to sectional representation, we covered several ploidy levels. In total, we included 21 diploid, one triploid, five tetraploid, four hexaploid, and one octoploid species. The samples were collected mainly in Central Europe, however, additional samples from Spain, United Kingdom, Northern Europe, as well as the United States were included. Species were determined after Skvortsov (1999), Argus (2010), and Hörandl et al. (2012). Leaves were dried in silica gel and herbarium voucher specimens were deposited in the herbarium of the University of Goettingen (GOET) and the University of South Bohemia. For phylogenetic analyses, we integrated 20 available plastomes from Genbank (Supplementary Table 1).

**Table 1:** Plant material including taxonomic classification and origin.

**Species**	**Subgenus**	**Section**	**Ploidy**	**Sample ID**	**Origin**
*Salix appendiculata*	*Chamaetia/Vetrix*	*Vetrix* subs. *Vulpinae*	2x	NW17.021	Austria
*Salix acutifolia*	*Chamaetia/Vetrix*	*Daphnella*	2x	ACU 1	Czech Republic
*Salix aurita*	*Chamaetia/Vetrix*	*Vetrix* subs. *Leaves*	2x	NW17.041	Austria
				AUR4	Czech Republic
*Salix bicolor*	*Chamaetia/Vetrix*	*Phylicifoliae*	3x	BIC3	Austria
*Salix breviserrata*	*Chamaetia/Vetrix*	*Myrtosalix*	2x	EH 10508	Spain
				BRE15	Austria
*Salix caesia*	*Chamaetia/Vetrix*	*Helix*	4x	CAE1	Austria
*Salix caprea*	*Chamaetia/Vetrix*	*Vetrix* subs. *Leaves*	2x	CP03	UK
				CAP2	Czech Republic
*Salix cinerea*	*Chamaetia/Vetrix*	*Vetrix* subs. *Leaves*	4x	NW17.082	Austria
				CIN1	Czech Republic
*Salix daphnoides*	*Chamaetia/Vetrix*	*Daphnella*	2x	DAP1	Czech Republic
*Salix eleagnos*	*Chamaetia/Vetrix*	*Cabae*	2x	EH 10495	Spain
*Salix foetida*	*Chamaetia/Vetrix*	*Villosae*	2x	FOE11	Austria
*Salix glabra*	*Chamaetia/Vetrix*	*Glabrella*	6x	GLA2	Austria
*Salix glaucosericea*	*Chamaetia/Vetrix*	*Glaucae*	8x	GSR7	Austria
*Salix hastata*	*Chamaetia/Vetrix*	*Hastatae*	2x	HAS3A	Austria
*Salix helvetica*	*Chamaetia/Vetrix*	*Villosae*	2x	1/2014	Switzerland
				HEL7	Austria
*Salix herbaceae*	*Chamaetia/Vetrix*	*Retusae*	2x	HER5	Austria
*Salix lapponum*	*Chamaetia/Vetrix*	*Villosae*	2x	LAP1	Czech Republic
*Salix mielichhoferi*	*Chamaetia/Vetrix*	*Nigricantes*	6x	MIE5	Austria
*Salix myrsinifolia*	*Chamaetia/Vetrix*	*Nigricantes*	6x	NW17.054	Austria
				MYS5	Austria
*Salix myrtilloides*	*Chamaetia/Vetrix*	*Myrtosalix*	2x	MYR1	Czech Republic
*Salix rosmarinifolia*	*Chamaetia/Vetrix*	*Incubaceae*	2x	ROS3	Czech Republic
*Salix reticulata*	*Chamaetia/Vetrix*	*Chamaetia*	2x	EH 10397	Italy
				RTI1	Austria
*Salix retusa*	*Chamaetia/Vetrix*	*Incubaceae*	6x	RET6	Austria
*Salix serpillifolia*	*Chamaetia/Vetrix*	*Incubaceae*	2x	SER22	Austria
*Salix silesiaca*	*Chamaetia/Vetrix*	*Vetrix* subs. *Vulpinae*	2x	SIL22	Czech Republic
*Salix sitchensis*	*Chamaetia/Vetrix*	*Sitchenses*	2x	NW18.046	California, USA
*Salix viminalis*	*Chamaetia/Vetrix*	*Vimen*	2x	VIM1	Czech Republic
*Salix waldsteiniana*	*Chamaetia/Vetrix*	*Villosae*	2x	WAL31	Austria
*Salix triandra*	*Salix* s.l.	*Amygdalinae*	2x	TRI4	Czech Republic
*Salix alba*	*Salix* s.l.	*Salix*	4x	EH 10431	Germany
				ALB8	Czech Republic
*Salix fragilis*	*Salix* s.l.	*Salix*	4x	FRA2	Czech Republic
*Salix pentandra*	*Salix* s.l.	*Pentandrae*	4x	EH 10470	Finland
				PEN3	Czech Republic

*Herbarium vouchers of collected samples are deposited at the herbarium of the University of Goettingen (GOET). Detailed information is given in Supplementary Table 1*.

### Genome Skimming and Reference-Based Mapping

The DNA of all samples was extracted using the Qiagen DNeasy Plant Mini Kit following instructions from the manufacturer (Valencia, CA). After quality check, the DNA of 12 samples was sent to NIG - NGS Integrative Genomics Core Unit of the University Medical Center Göttingen (UMG) (https://www.humangenetik-umg.de/en/research/nig/) for library preparation and sequencing. About 1 μg DNA of each sample was used for library preparation using the PCR FreeDNA Sample Prep Kit (Illumina) followed by the Illumina TruSeq PE Cluster Kit. The 12 samples were barcoded and multiplexed. Whole genome shotgun sequencing was performed on one lane of an Illumina HiSeq 2500 platform producing 2 × 150 bp paired end reads. With this initial test set, we wanted to assess the utility of whole genome skimming for *Salix* plastome reconstruction. The sequencing libraries for the remaining samples were generated using the NEBNext® DNA Library Prep Kit following recommendations of manufacturer. The NEBNext® Multiplex Oligos for Illumina kit was used to add indices to each sample and to enrich the libraries via PCR using P5 and indexed P7 oligos. The PCR products were then purified (AMPure XP system). Whole genome shotgun sequencing was carried out on a Novaseq 6000 platform producing 2 × 150 bp paired end reads. The quality of the resulting sequencing reads was checked with FastQC v.0.10.1 (Andrews, 2010), and the reads were assembled *de novo* for a total of 36 samples using the software Fast-Plast v.1.2.8 (McKain and Wilson, 2017) under its default settings. The minimum coverage was 0.25 of the average coverage across the respective plastome. For five samples, Fast-Plast was not able to assemble the complete plastome. It is known that fragments of the plastome were transferred to the nuclear genome in *Salix* (see Huang et al., 2014). These pseudo-copies might cause problems in a *de novo* plastome assembly approach based on deep sequencing data. To obtain plastomes for the five samples, we utilized a “mapping-to-reference” approach to receive the respective plastomes. For the reference-based assembly, we used Geneious vR11 2020.2.4 (http://www.geneious.com, Kearse et al., 2012) as described in Ripma et al. (2014). The reads were mapped to the plastome of *S. purpurea* [Genbank accession NC026722].

The annotation of plastomes was done using CPGAVAS2 (Shi et al., 2019) with default settings applying the dataset containing 2,544 reference plastomes. The results were checked and edited with Geneious R11 2020.2.4 (www.geneious.com).

### Phylogenetic Analyses

For final phylogenetic analyses, the sequences of the 41 produced plastomes were combined with 20 available *Salix* plastomes from Genbank resulting in a dataset comprising 61 samples (for details, see Supplementary Table 1). Complete plastid genomes were aligned as a single sequence with MAFFT v3 (as implemented in Geneious R11) by applying the automatic algorithm selection with a gap open penalty of 1.53 and an offset value of 0.123. One inverted repeat (IR) copy was excluded from the alignment to avoid double weighting of identical information. Because the overall variation was low, especially within the *Chamaetia/Vetrix* clade, the effects of misaligned regions in the subsequent tree topology could be pronounced (Parks et al., 2012; Duvall et al., 2020). Therefore, the alignment was optimized using Gblocks 0.91b (Castresana, 2000) with default settings (minimum number of sequences for a conserved position set to 25, minimum number of sequences for a flank position set to 40, maximum number of contiguous non-conserved positions set to 8, and minimum length of a block set to 10). The allowed gap position was set to “none” and “all,” respectively, and the results of both approaches were compared. The resulting alignments were extracted in PHYLIP format and used as input for Maximum Likelihood analysis using the general time-reversible (GTR)+Γ model of nucleotide substitution implemented in RAxML (Randomized Axelerated Maximum Likelihood) v.8.2.4 (Stamatakis, 2014). We conducted for each ML analysis a rapid bootstrapping (BS) analysis with 100 replicates. Resulting trees were obtained in FigTree v1.4.3 (Rambaut, 2014).

### Haplotype Networks

Next to phylogenetic tree reconstruction, we used the plastome data to calculate haplotype networks with TCS v1.21 (Clement et al., 2000). Due to the large genetic distance between the subclades, we conducted two separate analyses without out-group: one for the closely related *Chamaetia/Vetrix* clade and one for subgenus *Salix* s.l. We used only coding regions to avoid homoplasy in the data set. Gaps were treated as missing data.

### Statistical Tests

A geographic clustering rather than a taxonomic clustering is frequently observed in plastid-based studies (Gitzendanner et al., 2018). To test for this trend in our data, we correlated the genetic distance with the geographic distance of the included samples. We derived the genetic distance matrix from the branch lengths in the observed RAxML tree of the complete plastome dataset using the R package ape 5.0 (Paradis and Schliep, 2019). We calculated the geographic distance matrix based on the global positioning system (GPS) coordinates of our samples. For Genbank samples without detailed information on sampling localities, we used the distribution center of the species or the location of the institute that performed the analyses instead. We then correlated the two matrices using a Mantel test based on Pearson's product-moment correlation with 999 permutations in the R package vegan 2.5 (Oksanen et al., 2019). We performed the analysis with the full dataset and with subg. *Salix* and subg. *Chamaetia/Vetrix* clades separately. Furthermore, in the case of subg. *Chamaetia*/*Vetrix*, we also performed an analysis excluding its basal members (*S. arbutifolia, S. rorida, S. magnifica*, and *S. oreinoma*) that showed plastomes most divergent from the rest of the subgenus. All analyses were performed in R 3.6.1 (R Core Team, 2019).

To analyze if plastid genes are under selection, we calculated gene-wise ω (dN/dS ratios = non-synonymus vs. synonymous substitutions) with codeML implemented in paml version 4.8 (Yang, 2007). We used the model = 0 option, i.e., a single omega for the whole tree. We extracted the coding sequences (=CDS) of 72 genes (Supplementary Table 2) out of 78 genes in total and used the alignments and the RAxML tree of the complete sample set as input. Annotations of Genbank accessions were not complete in all cases and for statistical reasons we included only genes that were present/annotated in at least 60 of the 61 samples for this test.

### Comparison to Nuclear RAD Sequencing Data of a Comparative Subset

To evaluate the phylogenetic resolution and topology of the plastome phylogeny, we compared 10 samples of our plastome data to a comparative sampling of already published RAD sequencing data. The RAD sequencing data are available at Genbank (Bioproject PRJNA433286). Previous RAD sequencing studies included two to four individuals per species and rendered species as monophyletic lineages (Wagner et al., 2018, 2020), and hence we used only one representative sample per species for the RAD sequencing analysis here. *Salix triandra* was used as an out-group in both datasets. For the comparison, we used a subset of 10 representative plastomes of the *Chamaetia/Vetrix* clade. We used the same accessions in both datasets whenever possible. However, in two cases, due to the low amount of extracted DNA, we substituted the species in the RAD analysis with another individual of the same species. The reduced RAD sequencing set was analyzed with ipyrad v7.24 (Eaton and Overcast, 2016) using the same settings as described in Wagner et al. (2018). The minimum number of samples sharing a locus was set to 4, the maximum number of single nucleotide polymorphism(s) SNP(s) per locus was set to 20, and the maximum number of indels per locus was set to 8. With respect to the mixed ploidy of the dataset, we used a maximum of four alleles in the settings of the ipyrad pipeline (for more details see Wagner et al., 2020). Maximum Likelihood analyses for both the concatenated RAD loci as well as the plastomes were performed as described above.

## Results

### Plastome Reconstruction

The shotgun sequencing revealed an average of 71.65 Mio raw paired reads per sample. An average of 6.86 Mio paired reads mapped to the plastome. The average coverage was 7,733 reads. The plastome lengths varied between 155,414 bp (*S. mielichhoferi*) and 160,386 bp (*S. myrtilloides*). Length variation was due to one large insertion at the margin of the inverted repeat (IRb) that was observed in 21 samples, two large indels (>200 bp) in *S. triandra* and species of subg. *Salix*, several smaller indels (2–80 bp), and repetitive motifs (SSRs, tandem repeats). All obtained plastomes showed the typical tetrapartite structure of two IRs separating the small single-copy (SSC) region from the large single-copy (LSC) region. They contained 78 protein coding genes, 30 tRNAs, and 3 rRNAs. The order of genes was identical in all newly assembled plastomes. The annotated plastomes were uploaded to Genbank, their respective accession numbers (MW435413–MW435453) are provided in Supplementary Table 1.

### Phylogenetic Reconstructions

The plastome sequences presented here were aligned together with 20 available *Salix* plastomes from Genbank. The initial alignment of the complete plastomes of 61 samples had a length of 141,081 bp and after trimming of one IR and alignment optimization with Gblocks retained a length of 129,052 bp. The concatenated alignment of coding regions (CDS) had a length of 68,211 bp. The length of the edited alignment for the *Chamaetia/Vetrix* clade was 128,608 bp and 68,009 bp for the extracted coding regions. The lengths were 128,403 bp and 68,311 bp for subgenus *Salix*, respectively. The variation observed in the complete alignment was 1.68 and 0.72% in coding regions. Within the *Chamaetia/Vetrix* clade, 0.74% of sites were variable and we observed 0.41% variability in the alignment of extracted coding regions. Within subgenus *Salix*, we observed 0.64% variability and 0.35% of variable sites for CDS, respectively. A statistical comparison of the different alignment editing approaches is provided in Supplementary Table 3.

### Relationships of Genus *Salix* Based on Plastome Data

**Figure 1:**
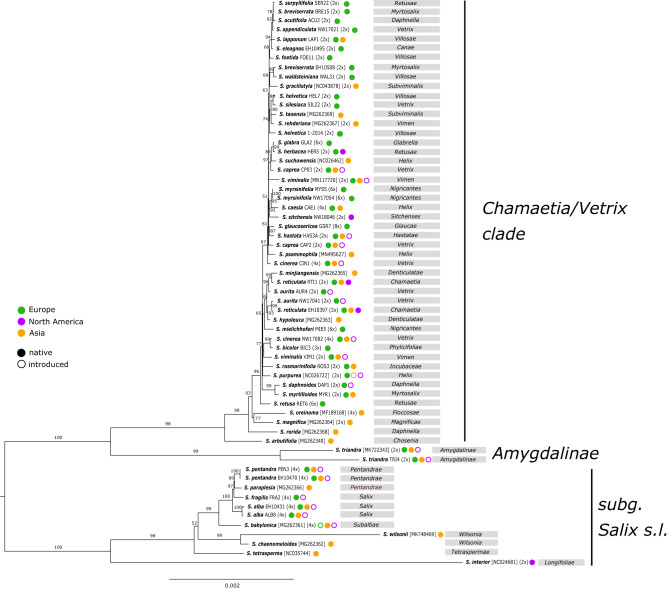
RAxML phylogeny based on complete plastomes of 61 samples representing 50 *Salix* species. Sample IDs or Genbank accession numbers for Genbank samples as well as ploidy levels are indicated behind species names. Bootstrap support values are given above branches. Geographic distribution of species is indicated behind sample names according to color coding. The taxonomical affiliation into sections is given in gray boxes. The two main clades are illustrated according to their subgeneric classification.

The observed phylogenetic tree based on complete plastomes showed a clear separation of the subgenus *Salix* s.l. (tree willows) (BS 100) and the *Chamaetia/Vetrix* clade (shrub willows) plus *S. triandra* (BS 100) (Figure 1). Both accessions of *S. triandra* (BS 99) were found to be sister to the well-supported *Chamaetia/Vetrix* clade (BS 98). Within the *Chamaetia/Vetrix* clade, the observed resolution was low, indicated by short branches and no or low BS support for most branches. *Salix arbutifolia* was in sister position to the remaining samples of *Chamaetia/Vetrix* (BS 100), followed by the Asian species *S. rorida*, and a clade comprising *S. magnifica* and *S. oreinoma* (BS 71). The remaining samples formed a well-supported clade (BS 100) with *Salix retusa* (RET6) at an early diverging position. The two accessions of *S. myrsinifolia* grouped together with high support (BS 100), while all other species with more than one sample appeared polyphyletic. Additionally, the sectional classification was not reflected by the phylogeny. No geographical pattern was observed, e.g., *Salix sitchensis* from California was shown to be closely related to European *S. myrsinifolia* and *S. caesia* (BS 92). Asian *S. gracilistyla* appeared in close relationship to *S. waldsteiniana* and *S. breviserrata*, both occurring in the European Alps. However, resolution within this clade was extremely low. Within the subg. /*Salix*/ clade, *S. babylonica* was in sister relationship to a subclade (BS 100) containing the European tree species (*S. alba, S. pentandra*, and *S. fragilis)*, and *S. paraplesia* from China. The North American willow *S. interior* was shown to be situated on a long branch and in sister position to the remaining samples of subg. *Salix*.

### Haplotype Networks

We calculated haplotype networks of the two subclades based on coding regions (CDS). For subg. *Salix*, the haplotype network displayed the same clades as the RAxML tree (Supplementary Figure 1). The close relationships of tree willows collected in Europe were reflected by a central empty haplotype that was not occupied by any included species. Both *S. alba* accessions were only one mutational step apart from each other, both accessions of *S. pentandra* shared an identical haplotype. *Salix paraplesia* showed close relationships to *S. alba, S. fragilis*, and *S. pentandra*. The remaining Asian species were up to 92 mutational steps distinct from the central haplotype connecting the European tree willows plus *S. paraplesia*.

**Figure 2:**
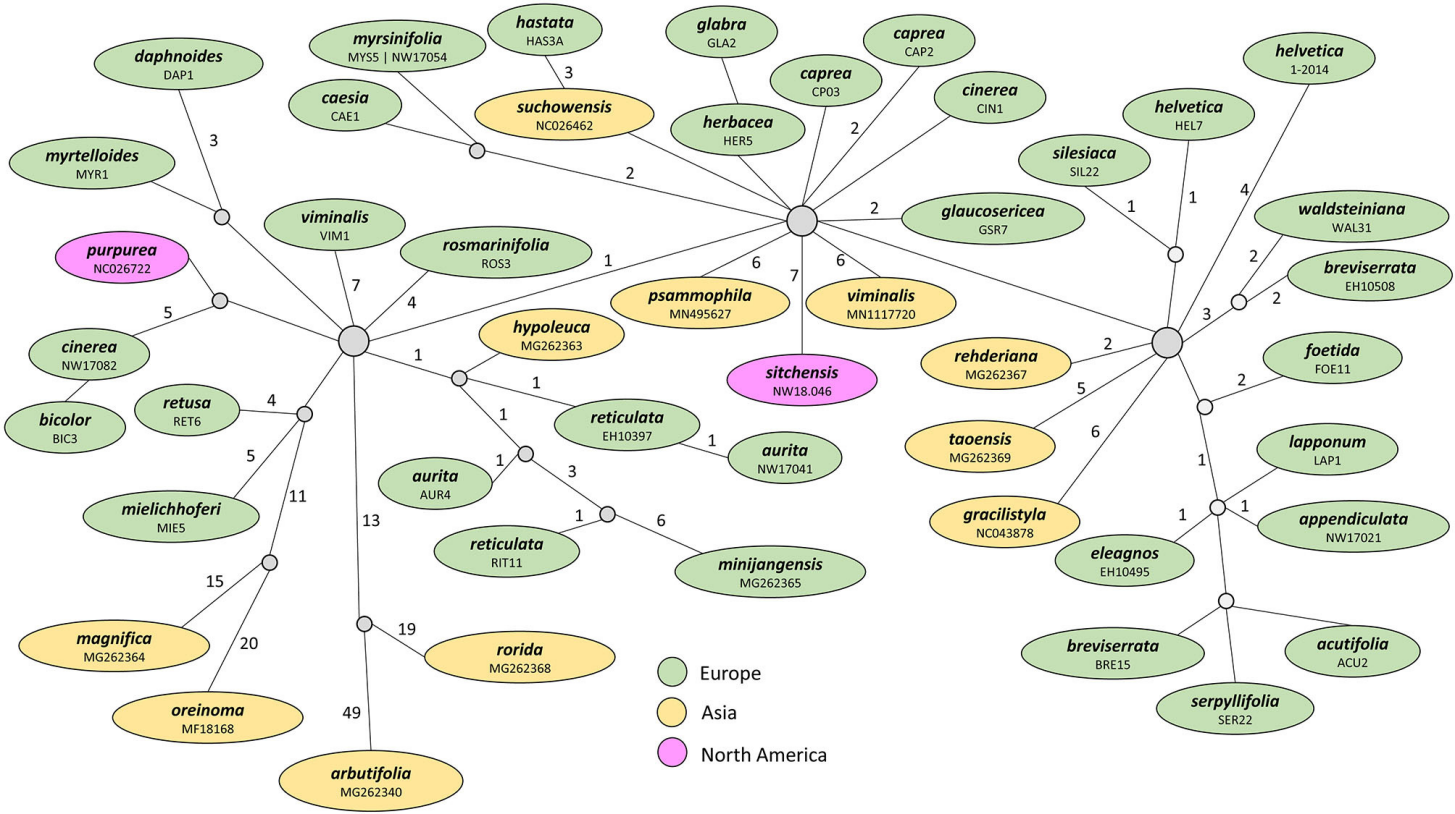
Haplotype network of 48 samples representing 42 species of the *Chamaetia/Vetrix* clade based on CDS regions. Each circle represents a unique haplotype, the number of mutational steps between haplotypes are indicated by numbers above branches. The applied color-coding reflects the geographic origin of samples. Both accessions of *S. myrsinifolia* share an identical haplotype. The colors highlight central haplotypes and closely related groups.

The haplotype network for the *Chamaetia/Vetrix* clade based on coding regions revealed that both accessions of *S. myrsinifolia* share the same haplotype. All other included samples showed a unique haplotype. Samples of the same species but from different localities did not group together, e.g., *S. caprea* from the United Kingdom was three mutational steps apart from *S. caprea* collected in the Czech Republic. Overall, the network showed three main groups that were connected via central haplotypes that were not occupied by included samples (Figure 2). Neither a geographical nor a taxonomical pattern was reflected.

### Statistical Tests

The Mantel tests revealed correlation of geographic and genetic distance in the case of the whole dataset (*r* = 0.1705 *p* = 0.018), for the subgenus *Salix* (*r* = 0.5025, *p* = 0.001), and for the whole subgenus *Chamaetia/Vetrix* clade (*r* = 0.2739, *p* = 0.018). When we excluded the early branching lineages of the *Chamaetia/Vetrix* clade, the significant correlation disappeared (*r* = 0.0619, *p* = 0.259).

The dN/dS ratios (non-synonymous vs. synonymous substitutions) revealed purifying selection for the majority of genes (ω values <1). Four genes showed ratios of positive, i.e., diversifying selection (*rpl*2, *rpl*16, *rps*15, and *ycf* 1). A complete list of gene-wise statistics and ω values is given in Supplementary Table 2.

### Comparison of Plastome and RAD Sequencing Data

**Figure 3:**
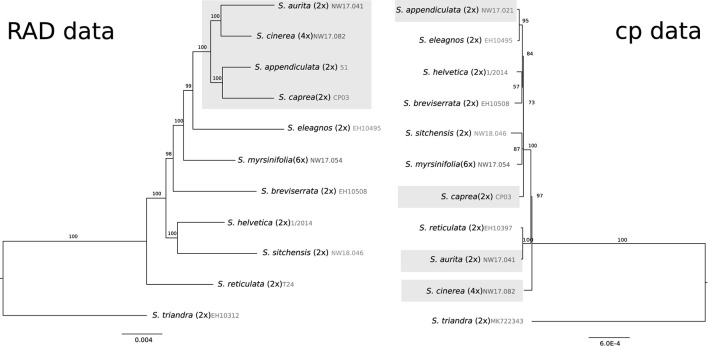
Comparison of Maximum Likelihood phylogenies of a subset of 10 species of the *Chamaetia/Vetrix* clade with *S. triandra* as out-group based on 57,084 RAD sequencing loci **(left)** and whole plastome data **(right)**. RAD sequencing data from Wagner et al. (2018, 2020). Bootstrap support values are given above branches. The ploidy level and accession numbers are indicated behind species names, the members of section *Vetrix* are highlighted in gray.

We compared the resulting phylogeny of 10 plastomes of the *Chamaetia/Vetrix* clade to published nuclear RAD sequencing data of the same subset of species. *Salix triandra* was used as an outgroup. The resulting plastome alignment of 130 kbp showed 0.5% variable sites. The RAD sequencing alignment of 57,084 concatenated RAD sequencing loci had a length of 4,669,722 bp and showed 8.05% variable sites. The alignment contained 26.3% missing data. The observed phylogeny based on RAD sequencing data was in accordance with formerly published data based on more samples (Wagner et al., 2018, 2020, 2021) (Figure 3). *Salix reticulata* was in sister position to the remaining species. Species belonging to section *Vetrix* formed a well-supported monophyletic group. The North American species *S. sitchensis* was situated in sister relationship to *S. helvetica*. The plastid phylogeny of the same subset showed tetraploid *S. cinerea* in sister position to the remaining species (Figure 3). The members of section *Vetrix* did not form a monophylum but occurred at different positions in the tree (highlighted in Figure 3). *Salix sitchensis* was in sister position to hexaploid *S. myrsinifolia*. The dwarf shrub *S. breviserrata* was found to be closely related to the Swiss willow *S. helvetica*. Overall, the branches were very short, however, the bootstrap values showed moderate to good support for the observed topology.

## Discussion

In 2014, Percy et al. proclaimed the “spectacular failure of barcodes for willows.” In contrast to single markers, we sought to analyze the variability of complete plastomes and their utility for phylogenetic analyses. We included 41 newly assembled plastomes and present here the first comprehensive study on plastome evolution on a subgeneric level within *Salix*. Compared to other angiosperms, our results reveal that the sequence variation in genus *Salix* is very low. Wu et al. (2015) observed 7.8% variable sites in their combined four-cp-marker set. However, the authors included next to *Salix* also samples of *Populus* and *Dovyales* as outgroups, which were responsible for most of the observed variation. To compare the results in detail, we extracted the four specific loci (*mat*K, *rbc*L, *atp*B-*rbc*L, and *trn*D-*trn*T) of our dataset and revealed 3.2% variable sites for the combined loci and complete sampling, but only 0.4% for the *Chamaetia/Vetrix* clade and 2.4% for subg. *Salix*, respectively (see Supplementary Table 4).

### Low Mutation Rates and Effective Repair Mechanisms in Coding Regions

Our alignment of the complete plastomes shows only 1.64% variable characters. Our alignment of all extracted coding regions shows only a variation of 0.72%. Genic regions of plastomes evolve with only about a third or half the rate of the nuclear genome (Wolfe et al., 1987). It is still unknown, why plant organellar genes have lower mutation rates than nuclear genes, but possible explanations include differences in replication enzymes, replication fidelities, mismatch repair, and low rates of genetic exchange (Gaut et al., 2011). Our results show a high degree of purifying selection in the protein coding genes of the plastome (Supplementary Table 2). The elimination of deleterious mutations might also affect linked sites and thus decrease the overall genetic variability (Charlesworth et al., 1993). Because the complete plastome can be treated as one single haplotype, this might be an explanation of the observed low variability. Next, recombination is a driver of purifying selection and can also happen between and among organelles (Bock et al., 2014). However, we assume that gene conversion might be a strong mechanism acting toward purifying selection in our dataset (Wolfe and Randle, 2004). Gene conversion is known from non-recombining systems, e.g., mitochondrial genomes (Mower et al., 2010) and nuclear genomes of ancient asexual animals (Flot et al., 2013). However, the elimination of deleterious mutations by gene conversion has also been proposed in plastid genomes (Khakhlova and Bock, 2006).

An overall low genetic divergence also occurs in the nuclear genomes of willows. Single barcoding regions like internal transcribed spacer (ITS) or single genes like *rbc*L and *mat*K have failed to resolve interspecific relationships (Leskinen and Alström-Rapaport, 1999; Lauron-Moreau et al., 2015). Instead, thousands of nuclear RAD sequencing loci were required to resolve species-level relationships in the *Chamaetia/Vetrix* clade (Wagner et al., 2018, 2020, 2021; He et al., 2021b). Based on our results, as well as on previous studies, we infer generally low mutation rates in willow genomes, considering the relatively high age of the genus (up to 43.8 Ma; Wu et al., 2015). Efficient regulation of intracellular oxidative stress resulting from photosynthesis and respiration might avoid DNA damage and reduce frequencies of non-homologous DNA repair processes, which is generally a major source for mutagenesis (Friedberg and Meira, 2006). Willows are rich in antioxidants, especially in phenolics and other typical chemical compounds known for the regulation of redox homeostasis (Hörandl et al., 2012; Jia et al., 2020; Piatczak et al., 2020). Their hypothetical role in the observed low mutation rates would need to be tested. However, it is remarkable that a low mutation rate (c. one-sixth of *Arabidopsis*) has also been observed in nuclear, plastid, and mitochondrial genomes of poplar (Tuskan et al., 2006), the sister genus of *Salix*, which is similarly rich in phenolics, such as salicylates, tannins, or flavonoids (Palo, 1984).

### Variable but Not Informative: Rapidly Evolving Non-coding Regions

In our dataset, we observed some length variation based on insertions/deletions resulting from sequence duplications in non-coding regions of the plastome, which is in the range of similar studies (Huang et al., 2017; Zhang et al., 2018; Li et al., 2019). Most of the observed variable characters occurred in rapidly evolving, non-coding parts of the plastome, as SSRs and other repetitive regions (Zhang et al., 2018; Li et al., 2019). Next to that, the haplotype network of the *Chamaetia/Vetrix* clade revealed mainly synonymous and non-directional mutations in coding regions (Figure 2). Homoplasy might be introduced by plastid haplotype polymorphism within and among individuals, resulting in paralogous copies (Wolfe and Randle, 2004). Further, the non-directional signal of mutations might lead to conflicting signals in the phylogeny (Parks et al., 2012; Duvall et al., 2020). Because the overall variability is very low, this effect might be even stronger within shrub willows. However, both the effects, low variation and non-directional signal, lead in combination to a non-resolved tree, especially in the *Chamaetia/Vetrix* clade. Interestingly, the effects in subgenus *Salix* seem to be less significant. Despite similar levels of variability, the topology of the subclade is much better resolved. This is in accordance with previous phylogenetic studies (Percy et al., 2014; Lauron-Moreau et al., 2015; Wu et al., 2015). Gene transfer from the plastome to the nucleus might give some additional explanation to the observed low variability (Bock and Timmis, 2008). For *Populus trichocarpa*, the transfer of the whole plastome to the nuclear genome was reported, while hints of transfer of single loci were also found in some *Salix* species (Huang et al., 2014). However, due to the lack of suitable genomes, we did not test for any transfer of plastid genes or larger portions of the plastome to the nuclear genome in our dataset. Nevertheless, the *de novo* assembly problems for five samples may have occurred due to the transfer of large portions of the plastome to the nucleus.

### Molecular Dating Opposed Hypotheses of Rapid Radiation or Rapid Range Expansion From Refugia

Another explanation for the observed low plastome variation, especially within the *Chamaetia/Vetrix* clade, might be a large radiation or a rapid range expansion from refugia after the last glacial maximum (Percy et al., 2014; Lauron-Moreau et al., 2015). In this scenario, recently evolved species would share identical haplotypes. Our data on shrub willows revealed a low amount of variation, but almost no identical haplotypes were observed. Additionally, lineage diversification clearly predates the Pleistocene glaciations; the age of genus *Salix* was estimated as 43.8 Ma, and that of the *Chamaetia/Vetrix* clade as 23 Ma, respectively (Wu et al., 2015). Next to the diversification time, the distinctiveness of the species based on morphology and nuclear phylogenies also oppose a postglacial rapid radiation as an explanation for low plastome variability (Wagner et al., 2018, 2020; He et al., 2021b). However, an older radiation followed by fragmentation and genetic drift cannot be ruled out completely.

### Differences Between Tree Willows (subg. *Salix*) and Shrub Willows (subg. *Chamaetia/Vetrix*)

Our comprehensive plastome phylogeny confirmed the differentiation into two distinct subgeneric clades (Wu et al., 2015; Huang et al., 2017; Zhang et al., 2018). An explanation for the split into two clades might be that species within subgenera and within sections hybridize more frequently than species between different subgenera (Hörandl, 1992). A recent study analyzed differences in sex determination systems in subg. *Salix* and the *Chamaetia/Vetrix* clade, which might be responsible for incompatibilities between the two subgeneric clades (He et al., 2021a). This would support our conclusion that the plastomes of the subgenera evolved more independently. Our data confirm the monophyly of species as well as the split of a New World and an Old World clade within subgenus *Salix* (Chen et al., 2010; Percy et al., 2014; Wu et al., 2015). The geographic pattern was further supported by the results of the Mantel test. Within the shrub willows, the somewhat isolated position of *S. arbutifolia* is in accordance with previous studies (Lauron-Moreau et al., 2015; Wu et al., 2015). The early branching Asian lineages corresponded to the Hengduan Mountain clade described in He et al. (2021b). Within the core *Chamaetia/Vetrix* clade, neither species-specific patterns nor support for previous sectional classification were found. The polyphyly of four species might be explained by homoplasy of plastid polymorphisms (see above).

### Comparison of Nuclear and Plastid Data

We compared nuclear RAD sequencing data with plastome data for a subset of 10 samples. So far, few studies have performed a statistical comparison of the two reduced representation methods we used here (Pham et al., 2017; Mu et al., 2020). Our comparison clearly shows that RAD sequencing is much more efficient in resolving relationships within *Salix* than plastome data. The included members of the section *Vetrix* showed a well-supported monophyletic group in the RAD sequencing dataset, which is in accordance with previously published data (Wagner et al., 2018, 2020, 2021). However, the same species were scattered over the non-resolved tree in the plastome phylogeny (Figure 3). Maternal inheritance of plastomes might explain the observed incongruence of nuclear (RAD sequencing) and plastome phylogenies. These discrepancies could be further explained by chloroplast capture, and in the case of polyploid *S. cinerea*, by an allopolyploid origin (Wagner et al., 2020). In willows c. 40% of species are polyploid (Suda and Argus, 1968), which means that frequent allopolyploidy could have a major impact on phylogenetic relationships. Different ancient hybrid origins will influence the backbone of the plastome tree and thus explain discordance between nuclear and plastid phylogenies. Further, more recent hybridization or introgression events, even if infrequent, could occur between distantly related species, transferring the few and randomly appearing plastome polymorphisms to different genomic backgrounds of species.

### The Lack of Any Biogeographical Pattern

In the presence of frequent hybridization as well as chloroplast capture, we would expect a biogeographic signal in the phylogeny and/or haplotype networks. Although our sampling represented mainly European species, it also included samples from several parts of Eurasia and North America. Based on our data, species of geographical proximity show distinct haplotypes while some species from separate continents share similar plastomes. For example, *S. sitchensis* from the West Coast of the United States shared a similar plastome with *S. caesia* and *S. myrsinifolia* from Europe. Over these huge distances, extant hybridization and frequent maternal gene flow via seeds is unlikely. However, within Eurasia, distribution areas of widespread species are often overlapping (see Skvortsov, 1999), and hybridization appears possible. Interestingly, the Mantel test revealed significant correlation of geographical and genetic distance in our dataset. This can be explained by the early branching lineages from China, which show quite distinct plastomes and might influence the results. When analyzing only the core clade of shrub willows, no correlation could be observed. This is in accordance with former results of Percy et al. (2014) who found correlation of geographical and genetic distance within the overall dataset, but no correlation within a large clade of shrub willows. The close relationship of Eurasian and North American shrub willow species in plastid-based phylogenies was also reported in Lauron-Moreau et al. (2015), who observed a large clade comprising boreo-arctic and montane to alpine species of both Eurasia and North America.

### Plastid Genes Under Selection

Percy et al. (2014) assumed that hybridization/ introgression alone could not explain the small number of shared haplotypes between a large number of distinct willow morphospecies. They assumed a trans-specific selective sweep as a potential reason for one dominant haplotype. The positively selected plastome would have been able to spread rapidly, probably aided by widespread species hybridizing with the local ones. Our haplotype network of the *Chamaetia/Vetrix* clade, however, does not support the predominance of one certain haplotype. Further, most of the observed variation occurred in non-coding regions, and within genes, mostly in synonymous sites. However, the scenario of a selective sweep would require a positive selection of plastid genes. Indeed, Huang et al. (2017) tested plastid coding regions and found seven genes under selection in Salicaceae. However, this is not reflected in our results. Most tested protein coding genes showed purifying selection (dN/dS <1). Only four genes (5.6%) showed signals of positive selection (*rpl*2, *rpl*16, *rps*15, and *ycf* 1). Interestingly, they differed from the selective genes found by Huang et al. (2017). The genes analyzed by Percy et al. (2014) (*mat*K, *rbc*L, *rpo*B, and *rpo*C1) were all under purifying selection with ω values far below one. Thus, our results based on more species strongly contradict the hypothesis of a selective sweep. In *rpl*2 and *rps*15, slight signals of positive selection were detected, but both are ribosomal genes. The signal of positive selection was strong (dN/dS > 1) only in the case of *ycf* 1. This large open reading frame was for a long time enigmatic and its function unknown. The gene *ycf* 1 has been predicted to have the highest nucleotide diversity (π) at the species level within angiosperm plastid genomes (Dong et al., 2012, 2015). More recently, it was shown that *ycf* 1 encodes for Tic214, a vital component of the *Arabidopsis* translocon on the inner chloroplast (TIC) membrane complex that is essential for plant viability (Kikuchi et al., 2013). However, in comparison to other plant genera, *ycf* 1 is relatively conserved and showed only 1.5% variability on the genus level within *Salix*. The lack of a predominant haplotype as well as the low number of genes under selection argue against the hypotheses of a selective sweep in willows.

## Conclusions

The observed plastome variation in willows is much lower than in other angiosperm lineages. Thus, even complete plastome data are unsuitable for phylogenetic reconstruction, DNA barcoding, and analyses of biogeographical history in shrub willows. Usual explanations for plastome evolution patterns do not fit our data. Instead, the willow plastomes seem to have been shaped by extremely low mutation rates due to efficient mechanisms preventing mutagenesis, and further, by reticulate evolution and non-specific, rather random polymorphisms resulting in homoplasy. Consequently, the observed plastomes are neither species-specific nor reflect geographical patterns. Our results provide a caveat on relying solely on plastid phylogenies, a common practice in plant systematics. Our study demonstrates the importance of examining the evolution of plastid genomes thoroughly before applying them to questions of plant systematics, especially in cases of widespread, hybridizing taxa with low evolutionary rates.

## Data Availability Statement

The datasets presented in this study can be found in online repositories. The names of the repository/repositories and accession number(s) can be found at: https://www.ncbi.nlm.nih.gov/genbank/, MW435413 - MW435453.

## Author Contributions

NW planned and designed research. NW, MV, and EH conducted fieldwork. NW and MV performed experiments and analyzed data. NW wrote the manuscript. MV and EH contributed to the manuscript. All authors contributed to the article and approved the submitted version.

## Conflict of Interest

The authors declare that the research was conducted in the absence of any commercial or financial relationships that could be construed as a potential conflict of interest. The reviewer RS declared a shared affiliation, with no collaboration, with one of the authors, MV, to the handling editor at the time of the review.

## Publisher's Note

All claims expressed in this article are solely those of the authors and do not necessarily represent those of their affiliated organizations, or those of the publisher, the editors and the reviewers. Any product that may be evaluated in this article, or claim that may be made by its manufacturer, is not guaranteed or endorsed by the publisher.
