# Supplementary material for: Highly Diverse Shrub Willows (Salix L.) Share Highly Similar Plastomes
Source: Front Plant Sci. 2021 Sep 3;12:662715. doi: 10.3389/fpls.2021.662715 (PMC8448165; doi:10.3389/fpls.2021.662715)
Supplement: Supplementary file 1 [file Data_Sheet_1.zip › Supplementary Figure 1.pdf]

Supplement information for

Highly diverse shrub willows (*Salix* L.) share highly similar plastomes

Natascha D. Wagner, Martin Volf, Elvira Hörandl

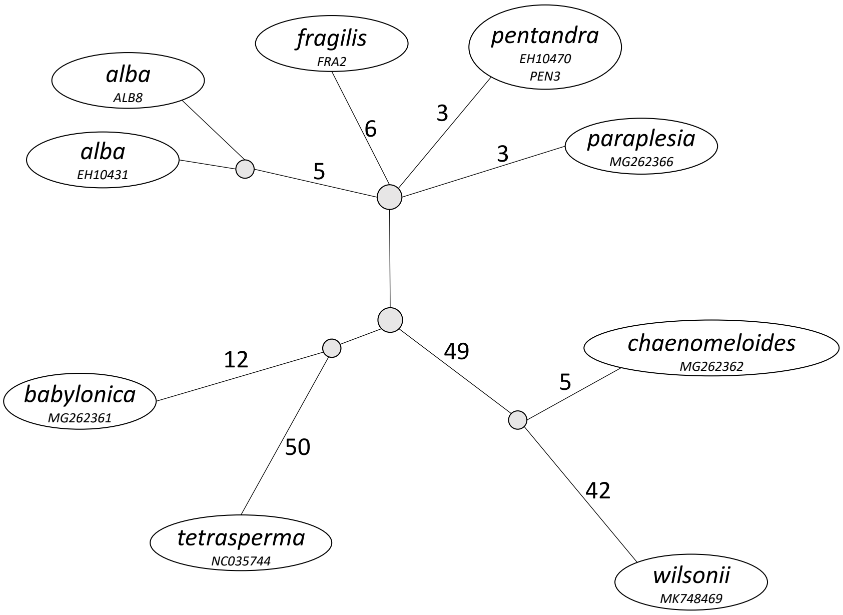

Fig. S1. Haplotype network of 10 samples representing 8 species of subg. *Salix* based on CDS regions. Number of mutational steps between individuals are indicated on branches. *Salix integra* was excluded because of the big genetic distance. Each circle represents a single haplotype, both accessions of *S. pentandra* share an identical haplotype.
