# Supplementary material for: Highly Diverse Shrub Willows (Salix L.) Share Highly Similar Plastomes
Source: Front Plant Sci. 2021 Sep 3;12:662715. doi: 10.3389/fpls.2021.662715 (PMC8448165; doi:10.3389/fpls.2021.662715)
Supplement: Supplementary file 1 [file Data_Sheet_1.zip › Supplementary Table 3.pdf]

Supplement information for

Highly diverse shrub willows (*Salix* L.) share highly similar plastomes

Natascha D. Wagner, Martin Volf, Elvira Hörandl

Supplement Table S3: Alignment statistics

Plastome datasets (all without IRa)	Edited alignment length [bp]	variability	Undetermined characters/ gaps
**Complete dataset (61 taxa)**			
Unedited	141,081	2,01%	8.41%
manually edited	131,869	1.51%	3.77%
Gblocks - Allgaps	129052	1,68%	1,23%
Gblocks - nogaps	121,139	0.55%	-
Extracted coding regions (CDS)	68,211	0.72%	0.35%
***Chamaetia/Vetrix (*50, incl. outgroup)**			
unedited	139,976	1,01%	7.56%
manually edited	130,201	0.74%	1.90%
Gblocks - Allgaps	128,853	0.92%	0.74%
Gblocks - nogaps	124,199	0.33%	-
Extracted coding regions (CDS)	68,009	0.41%	0.16%
***Chamaetia/Vetrix* (48, without outgroup)**			
Unedited	134415	0.85%	3.80%
manually edited	128,832	0.58%	1.43%
Gblocks - Allgaps	128608	0.74%	0.51%
Gblocks - nogaps	125368	0.28%	-
**Extracted coding regions (CDS)***	**67,927**	**0.32%**	**0.16%**
***Subg. Salix* (13, incl. outgroup)**			
Unedited	138,567	0.80%	7.02%
Manually edited	132,241	0.48%	4.38%
Gblocks – allgaps	128,354	0.64%	1.01%
Gblocks - nogaps	122,848	0.23%	-
Extracted coding regions (CDS)	68,311	0.35%	0.34%
***Subg. Salix* (10, without outgroup)**			
Unedited	134520	0.34%	4.16%
Manually edited	130,784	0.33%	2.11%
Gblocks - Allgaps	128403	0.30%	0.52%
Gblocks - nogaps	125603	0.11%	-
**Salix 10 CDS (without out, without interior)***	**68,270**	**0.18%**	**0.11%**

*used for TCS haplotype networks
